# Refractory Hypokalemia in Secretory Diarrhea Phenotype of Colonic Pseudo-Obstruction (Ogilvie's Syndrome)

**DOI:** 10.7759/cureus.32026

**Published:** 2022-11-29

**Authors:** Ahmad A Hazzazi, Saleh H Aloyouny, Akbar Saleem

**Affiliations:** 1 Internal Medicine, King Abdulaziz Medical City, Riyadh, SAU

**Keywords:** refractory, hypokalemia, secretory diarrhea, ogilvie's syndrome, colonic pseudo-obstruction

## Abstract

Colonic pseudo-obstruction is an acute non-obstructive colonic dilation associated with constipation or secretory diarrhea. The secretory diarrhea phenotype is associated with refractory hypokalemia that may require different interventions to treat. We present a case of a 51-year-old male who was admitted with a hemorrhagic stroke whose hospital course was complicated by severe abdominal distension, diarrhea, and hypokalemia. Initial investigations excluded infectious causes. Imaging confirmed colonic pseudo-obstruction. The hypokalemia was severe and refractory, requiring daily potassium replacement along with rectal tube decompression and spironolactone. Despite these interventions, the hypokalemia persists and requires nearly 100 days to resolve completely.

## Introduction

Colonic pseudo-obstruction (CPO) or Ogilvie's syndrome is a disorder in which the colon is abnormally dilated without mechanical obstruction or inflammation [1]. Many conditions have been associated with the development of CPO, including electrolyte imbalance, stroke, shock, myocardial infarction, major orthopedic surgeries, renal failure, organ transplant, chemotherapy, or opiate use [2]. Two phenotypes of CPO have been described, the classical CPO (C-CPO) and the secretory diarrhea CPO (SD-CPO). C-CPO is the most common phenotype and is associated with constipation. SD-CPO is the least common and is associated with diarrhea, profound hypokalemia, resistance to treatment, and increased mortality [3]. Treatment of the hypokalemia associated with SD-CPO is challenging and usually requires a prolonged period of therapy to resolve completely [4]. Herein, we present a case of refractory hypokalemia in SD-CPO to demonstrate to what extent it can be refractory and what measures can be used to treat it.

## Case presentation

**Figure 1:**
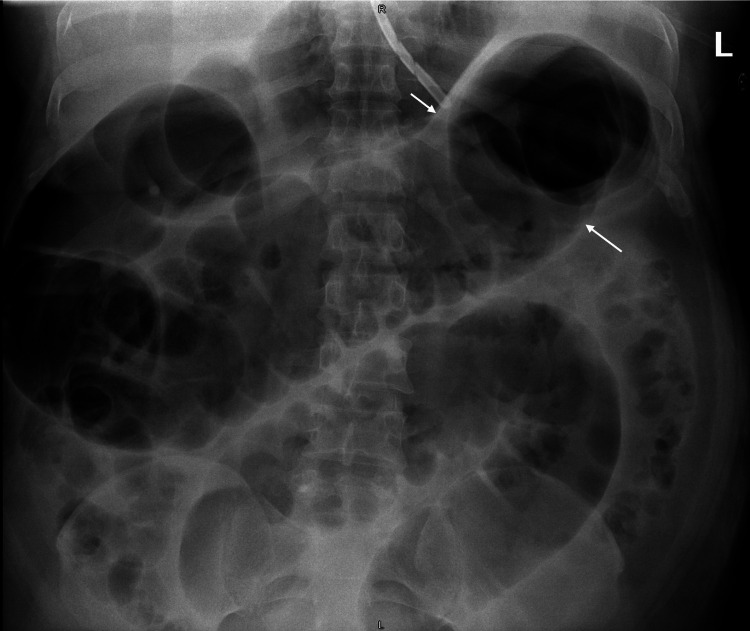
Abdominal x-ray shows colonic dilatation (White arrows)

A 51-year-old Indian male with a history of hypertension, chronic kidney disease, and stroke was found collapsed in his room and then brought to the emergency room (ER). Upon arrival, he was gasping and vomited twice; therefore, he was intubated by ER team to secure his airway. Initial examination revealed blood pressure 237/177 mmHg, Glasgow Coma Scale 3/15 with pinpoint pupils. Brain computerized tomography (CT) showed extensive pontine hemorrhage. The patient was admitted to the intensive care unit and managed by the neurology team. His hospital course was complicated by ventilator-associated pneumonia, which was treated with antibiotics. On the 10th day of admission, he developed abdominal distension and mild hypokalemia (K^+^ 3.3 mmol/L). Abdomen x-ray showed a distended colon with a diameter of around 10 cm (see figure 1), and potassium replacement was started.

On the 12th day, the patient developed watery diarrhea with a further decline in K^+^ level. Stool PCR was negative for *Clostridium difficile* infection. Stool microscopic examination and cultures were negative for other infectious causes. The diarrhea was persistent with worsening abdominal distension, so the patient was kept on frequent K⁺ replacement of around 60-90mEq per day.

On the 25th day, laboratory workup showed hypokalemia K^+ ^2.9 mmol/L, normal serum Magnesium 1.02 mmol/L, and hypernatremia 149mEq/L. Urine labs showed urine K^+^ 5.0mEq, and urine creatinine (Cr) 2.7 mmol/L. The urine K^+^/Cr ratio was less than 13mEq/g. Arterial blood gases showed normal anion gap metabolic acidosis. Stool K^+^ was high at 162.27mmol/L. These results were highly suggestive of gastrointestinal loss of potassium rather than renal loss.

**Figure 2:**
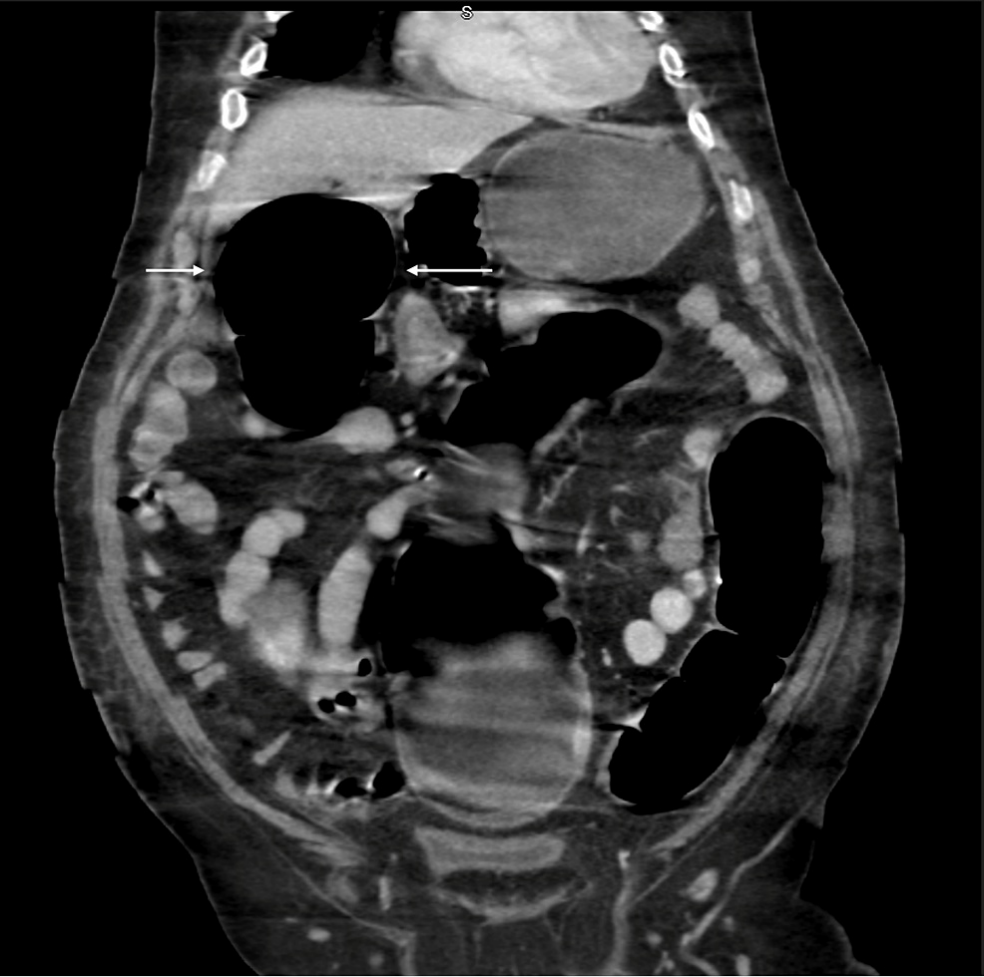
Abdominal CT in coronal view shows severe colonic dilatation (White arrows).

**Figure 3:**
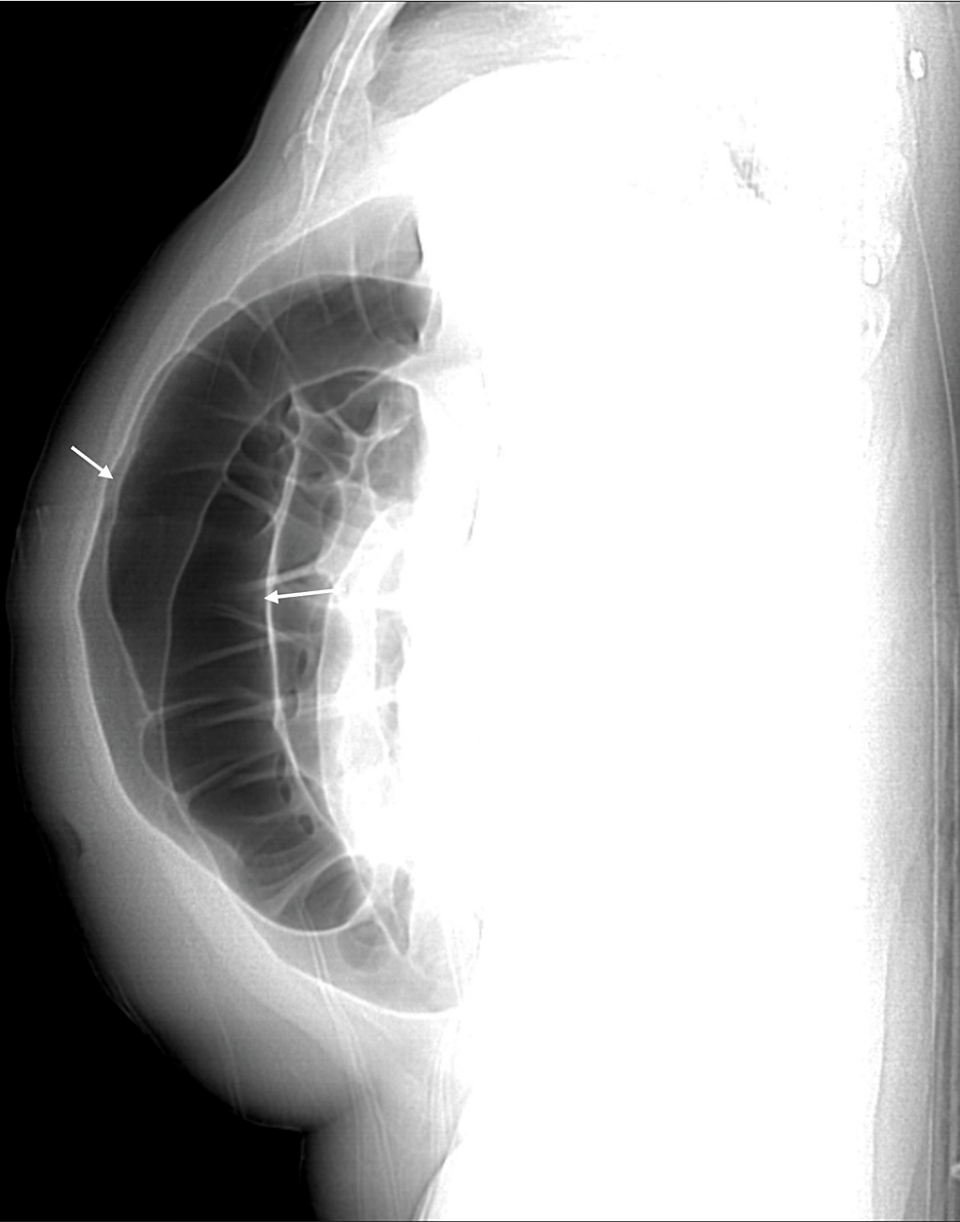
Abdominal CT in sagittal view shows severe colonic dilatation (White arrows).

**Figure 4:**
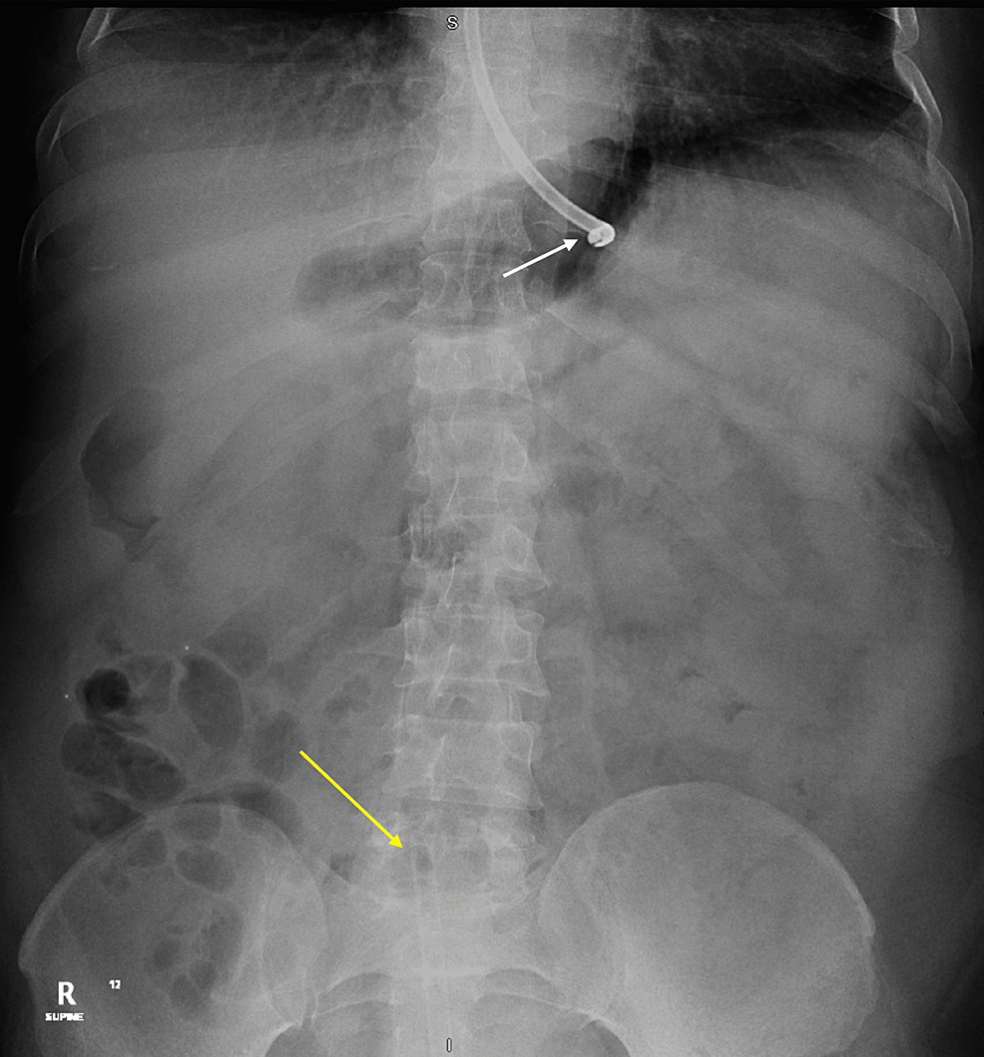
Abdominal x-ray shows resolution of colonic dilatation with Nasogastric tube (White arrow) and Rectal tube (Yellow arrow) in place.

One day later, abdominal CT was performed due to a severely distended abdomen and showed markedly diffuse colonic dilatation with no abrupt transition point or obstructing lesion (see figure 2, 3). Therefore, the diagnosis of colonic pseudoobstruction was made. The gastroenterology team was consulted, and they initially recommended electrolyte correction. However, the abdominal distension and the hypokalemia did not improve with electrolyte correction; thus, a rectal tube was inserted on day 40. After the insertion of the rectal tube, two liters of watery diarrhea was drained and the patient's abdomen was deflated. The rectal tube output was around 500-1000ml per day. In addition, spironolactone was started with a dose of 25mg/day, which later increased to 50 mg/day. Ten days later, the abdominal distension resolved, and the abdomen x-ray showed complete resolution of colonic dilatation (see figure 4). Unfortunately, multiple trials of rectal tube removal were unsuccessful due to the recurrence of colonic dilation. Three months later, the hypokalemia was corrected, and the patient maintained a stable serum K^+^ level without needing K^+^ replacement.

## Discussion

Severe hypokalemia is the most critical electrolyte abnormality associated with SD-CPO. It predisposes patients to severe complications that could be fatal. Thus, a better understanding of the pathophysiology can help treat and prevent complications. Patients with SD-CPO may have increased K^+^ excretion via the colon secondary to overexpression of the Big potassium (BK) channel in the colonic mucosa [4]. These voltage-gated channels transmit large amounts of K^+^ through the cell membrane [5]. It has been noted these channels are overexpressed in patients with end-stage renal disease as a protective mechanism to excrete K^+^ as the kidneys can no longer excrete K^+ ^[4,6]. Moreover, increased aldosterone secretion due to volume depletion stimulates these channels via the cAMP pathway [7]. As a result, secretory diarrhea pooling inside the distended bowel loops will be rich in K^+^. Additionally, increased β1-adrenergic stimulation to the Na⁺/K⁺ pump in the colonic mucosa has been linked to increased colonic K⁺ loss [3]. Based on these mechanisms, various ways of therapies have been utilized. One of these modalities is rectal tube decompression or decompressive colonoscopy, which is used for CPO management in general [8,9]. Another therapy is aldosterone antagonists, mainly spironolactone which acts by suppressing the colonic BK channels. Therefore, it reduces colonic excretion of K⁺, as well as decreases renal loss of K⁺. The first case to use spironolactone as part of the treatment of hypokalemia associated with SD-CPO was by Ram et al. in 2016 [7]. Subsequent case reports reported significant improvement in hypokalemia after the initiation of spironolactone [3,10,11]. Similarly, Somatostatin and its analog inhibit BK channels [12], leading to the improvement of hypokalemia in a patient with SD-CPO [13]. Furthermore, Neostigmine is one of the effective treatments for CPO [14]. It has a parasympathomimetic effect that increases intestinal motility and contractility. Consequently, it halts the process of colonic dilatation and prevents its recurrence [15]. It has been reported that hypokalemia can improve after the initiation of Neostigmine in patients with colonic pseudoobstruction [10]. Lastly, surgical interventions can be used as the last resort if conservative, pharmacological, and colonoscopic therapies are ineffective. However, it is associated with increased mortality [2]. Our patient's hypokalemia was secondary to high K⁺ losses in diarrhea, which was supported by high fecal K⁺ and normal urine K⁺ levels. It was refractory to K⁺ replacement and required prolonged time and different interventions to resolve. The recurrence of CPO in our patient was the main reason for hypokalemia's delayed resolution. Interestingly, CPO and hypokalemia can worsen each other, making the treatment challenging.

## Conclusions

SD-CPO is an acute condition that can lead to critical hypokalemia predisposing the patients to severe complications. This hypokalemia can be refractory and may require additional interventions to treat. Physicians should be aware of the risk factors, the pathophysiology of CPO, and the treatment options to avoid delays in management.
